# Identification and Validation of Reference Genes for Quantitative Real-Time PCR Normalization and Its Applications in *Lycium*

**DOI:** 10.1371/journal.pone.0097039

**Published:** 2014-05-08

**Authors:** Shaohua Zeng, Yongliang Liu, Min Wu, Xiaomin Liu, Xiaofei Shen, Chunzhao Liu, Ying Wang

**Affiliations:** 1 Key Laboratory of Plant Resources Conservation and Sustainable Utilization, South China Botanical Garden, Chinese Academy of Sciences, Guangzhou, P.R. China; 2 Key Laboratory of Pant Germplasm Enhancement and Specialty Agriculture, Wuhan Botanical Garden, Chinese Academy of Sciences, Wuhan, Hubei, P.R. China; 3 National Key Laboratory of Biochemical Engineering, Institute of Process Engineering, Chinese Academy of Sciences, Beijing, P.R. China; Key Laboratory of Horticultural Plant Biology (MOE), China

## Abstract

*Lycium barbarum* and *L. ruthenicum* are extensively used as traditional Chinese medicinal plants. Next generation sequencing technology provides a powerful tool for analyzing transcriptomic profiles of gene expression in non-model species. Such gene expression can then be confirmed with quantitative real-time polymerase chain reaction (qRT-PCR). Therefore, use of systematically identified suitable reference genes is a prerequisite for obtaining reliable gene expression data. Here, we calculated the expression stability of 18 candidate reference genes across samples from different tissues and grown under salt stress using geNorm and NormFinder procedures. The geNorm-determined rank of reference genes was similar to those defined by NormFinder with some differences. Both procedures confirmed that the single most stable reference gene was *ACNTIN1* for *L. barbarum* fruits, *H2B1* for *L. barbarum* roots, and *EF1α* for *L. ruthenicum* fruits. *PGK3*, *H2B2*, and *PGK3* were identified as the best stable reference genes for salt-treated *L. ruthenicum* leaves, roots, and stems, respectively. *H2B1* and *GAPDH1*+*PGK1* for *L. ruthenicum* and *SAMDC2+H2B1* for *L. barbarum* were the best single and/or combined reference genes across all samples. Finally, expression of salt-responsive gene *NAC*, fruit ripening candidate gene *LrPG*, and anthocyanin genes were investigated to confirm the validity of the selected reference genes. Suitable reference genes identified in this study provide a foundation for accurately assessing gene expression and further better understanding of novel gene function to elucidate molecular mechanisms behind particular biological/physiological processes in *Lycium*.

## Introduction

*Lycium* belong to the Solanaceae family and include seven Chinese species, *L. chinense* Miller, *L. ruthenicum* Murray, *L. truncatum* Y. C. Wang, *L. barbarum L.*, *L. cylindricum* Kuang et A. M. Lu, *L. truncatum* Y. C. Wang, and *L. yunnanense* Kuang. Of those, *L. barbarum* and *L. ruthenicum* have been extensively used as medicinal and functional foods in China for more than 2000 years. Several Chinese medicinal monographs depict their functions in nourishing the liver and kidney, enhancing eyesight, enriching blood, invigorating sex, reducing rheumatism, curing heart disease and correcting abnormal menstruation. These health-promoting phytochemical compounds, including anthocyanins and carotenoids, accumulate in *Lycium* fruits [1], [2]. At this time, anthocyanin biosynthesis is well known [3] and the anthocyanin regulatory model of BMW tricomplex, formed by **b**HLH, **M**YB, and **W**D40 transcription factors, has been established [4]. The BMW tricomplex is responsible for transcription of several anthocyanin structural genes, including *flavonoid 3′hydroxylase* (*F3′H*, EC: 1.14.13.21) and *flavonoid 3′5′hydroxylase* (*F3′5′H*, EC 1.14.13.88). All anthocyanin structural genes were recently isolated and characterized in *L. ruthenicum* [5]. In addition, petunidin derivatives account for 95% of the anthocyanins in *L. ruthenicum* fruits [1], suggesting that metabolic flux was largely introduced into the delphindin branch by F3′5′H enzymes while not into the cyanidin branch by the F3′H enzyme. In the anthocyanin pathway, F3′5′H enzymes compete with F3′H enzymes for the same substrate, dihydrokaempferol, and the anthocyanin pathway in *L. ruthenicum* fruit has been predicted (Fig. S1).

*L. barbarum* and *L. ruthenicum* are widely cultivated and distributed in Northwest China because they are drought-, alkaline-, and salt-resistant. These unique characteristics enable *Lycium* to prevent soil desertification and improve soil salinity/alkalinity, which is necessary for ecosystem protection and agricultural stability in remote areas of Northwest China. Recently, *SlNAC1* transcripts were reported to be increased in tomato (*Solanum lycopersicum*) roots under salt-stress [6]. Thus, *SlNAC1* was thought to be a salt stress-responsive gene marker. *Lycium NAC*, which is homologous to *SlNAC1*, is a candidate gene for investigating molecular mechanisms behind *Lycium* tolerance to salt stress.

Several fruit-specific genes, including *polygalacturonase* (*PG*) [7], [8] and *E8* [9], have been identified in the tomato and the *PG* gene encodes major cell wall degradation enzymes [8]. Previous studies document that *SlPG* transcripts initially appear at the onset of ripening and remain expressed during the ripening process, but they are not detected in unripe fruits, roots, or leaves [7], [8]. *SlPG* expression is attributed to the distal 3.4 kb of the 4.8 kb promoter sequence, which contains a 400 bp imperfect reverse repeat that shares high sequence similarity to the promoter sequence of the ripening-related *E8* [9]. Additionally, a positive cis-element (−806–443), responsible for expression in both inner and outer pericarp, and a negative cis-element (−1411–1150), restricting expression to the outer pericarp, were identified in the *SlPG* promoter [10]. Thus, *SlPG* is thought to have ripening-specific expression in tomato fruits.

Studies thus far in *Lycium* species have focused on phytochemical extraction [1], [2], [11] and medical usage of the extracts [12], [13]. Fewer studies have attempted to uncover the underlying molecular biosynthetic and regulatory mechanisms of these medicinal, phytochemical components. Thus, understanding gene expression patterns may offer clues of complex regulatory networks and help us identify genes relevant to novel biological processes such as salt-resistance, fruit ripening and anthocyanin biosynthesis in *Lycium*. To this end, we screened and evaluated candidate reference genes using quantitative reverse-transcription PCR (qRT-PCR) to measure expression across different samples.

An ideal reference gene should have stable expression in all tissues and under various experimental conditions. Housekeeping genes (HKGs) are usually presumed to be stable in this way, and they are often chosen as candidate reference genes. In the past, HKGs have been extensively used to evaluate gene expression by qRT-PCR without systematic experimental verification. Such universally-used HKGs include *elongation factor1-α* (*EF1α*) [14]–[17], *actin* (*ACTIN*) [14]–[17], *cyclophilin* (*CYC*) [14], [15], [17], *glyceraldehyde-3-phosphate dehydrogenase* (*GAPDH*) [14]–[16], *ubiquitin* (*UBQ*) [18], [19], *phosphoglycerate kinase* (*PGK*) [20], [21], *alpha-tubulin* (*TUA*) [14]–[16], *ubiquitin conjugating enzyme* (*UBCE*) [14], [16], and *s-adenosyl methionine decarboxylase* (*SAMDC*) [14], [16]. Unfortunately, ideal reference genes do not exist [22]–[25], so using an undefined HKG would not be prudent for normalizing gene expression, especially when only one HKG is used as a reference gene [23].

Several procedures, including geNorm [22] and NormFinder [26], were exploited to identify the optimal reference gene(s) stably expressed in a given set of tissues and experimental conditions. geNorm is defect-sensitive to co-regulated genes among candidates [22], which can be surmounted by NormFinder [26]. The principle difference between geNorm and NormFinder also results in discrepancy in ranking candidate genes [26]. Using these two procedures, an increasing number of reference genes have been identified in various species, including *Arabidopsis* [27], rice [28], potato [17], *Brachypodium* [16], flax [15], banana [14], and the common bean [29]. At this time, identification of stable reference genes has only been investigated in *L. barbarum* ripening fruit [18] but not in other *Lycium* species or tissues. Additionally, many stably expressing reference genes have been identified in fruit [14] and seeds [28], as well as reference genes that are responsible for different plant developmental stages [14], [15], [28], responses to biotic stress [14], [17], responses to abiotic stress such as cold [17], [28], [29], drought [29], salinity [17], [29], and responses to hormone treatment [14], [16].

In this study, with the purpose of identifying suitable reference genes for accurate evaluation of gene expression in ripening fruits or plants under salt stress. Thus, the expression stability of 18 candidate reference genes was calculated using geNorm and NormFinder procedures. Data show that candidate reference genes ranked by geNorm algorithm were similar to those defined by the NormFinder algorithm. Also, expression of the salt-responsive gene *NAC*, the fruit-specific gene *PG*, and several anthocyanin genes were normalized using the selected reference genes. The reference genes identified here will help researchers more precisely assess gene expression and better understand novel gene function to elucidate specific molecular mechanisms of particular biological/physiological processes in *Lycium*.

## Materials and Methods

### Plant Materials and Stress Treatments

Both *L. barbarum* and *L. ruthenicum* fruits were harvested from Zhongning County, Ningxia Hui autonomous region, P. R. China and Shihezi County, Xinjiang Uygur autonomous region, P. R. China, respectively. No specific permissions were required for these locations/activities. Fruit samples were divided into five specimens corresponding to five developmental stages (S1–S5) to identify fruit-specific candidate reference genes. In *L. ruthenicum* fruits, phenotypic changes of S1 fruits converting to S2 were from green to light pink. S3 fruits were dark purple while S4 fruits were black. Furthermore, black S5 fruits were fully expanded. In *L. barbarum* fruits, green S1 fruits turned light yellow S2 fruits. Also, variegated S3 fruits turned red S4 and red S5 fruits were matured and fully expanded. In addition, *L. ruthenicum* sepals, petals, stamens, pistils, roots, stems, and leaves were harvested to determine the stability of tissue-specific candidate reference genes. Of those tissues, roots, stems, and leaves were derived from forty-day old *L. ruthenicum* seedlings. For salt treatment, forty-day old seedlings of *L. barbarum* and *L. ruthenicum* were treated with 500 mM for 0, 0.5, 1, 2, 4, 8, and 16 h. Seedlings dissected into roots, leaves, and stems were sampled. Under 21–23°C growth conditions, forty-day old *L. barbarum* and *L. ruthenicum* seedlings were cultured under a photoperiod of 16/8 h (day/night). Samples were prepared in triplicate.

### Data Acquisition and Statistical Analysis

In this study, 17, 18, and 17 candidate reference genes were used to identify reference gene targets suitable for evaluating gene expression in different tissues, salt-treated seedlings, and developmental fruits of *L. ruthenicum*, respectively (Table S1). Also, 12 and 9 candidate reference genes were used to identify the reference gene targets suitable for evaluating gene expression in fruits and salt-treated roots of *L. barbarum*, respectively (Table S1). Candidate reference gene expression was defined as the number of cycles needed to reach a threshold fixed in the exponential phase of PCR (Cp) [30]. As suggested in algorithm manual, Cp values generated by LightCycle480 Detection System (Roche, USA) were transformed using the delta-Cp method. Resulting values were put into geNorm [22] and NormFinder [26] to measure gene expression. The geNorm procedure calculated the expression stability value (M) for each gene and the pairwise variation (V) of a certain gene compared with remains. Finally, all candidate reference genes were ranked according to their stability in the samples, and the optimal number of reference genes benefit for accurate normalization is suggested [22]. In contrast, NormFinder independently ranks the stability of candidate reference genes and it calculates not only the overall candidate reference gene variation but also the variation between sample subgroups of the sample set [26]. Statistical analyses were performed with ANOVA.

### Total RNA Extraction and Template Preparation

Total RNA was extracted from all samples using Trizol kit (Invitrogen, USA). The quality and amount was confirmed with 1% gel electrophoresis and Nanodrop, respectively. Only high quality RNA samples were used for subsequent analyses. Total RNA (1 µg) was reverse-transcribed using PrimeScript RT Reagent Kit with gDNA Eraser (DDR047, TaKaRa), which digested residual RNA sample DNA and reverse-transcribed in one step. cDNA templates were diluted and used (20 ng cDNA per reaction) for qRT-PCR.

### Primer Design, Verification of PCR Products and qRT-PCR

Several potential reference genes, including *ACTIN*, *EF-1α*, *GAPDH*, *UBQ*, *SAMDC*, *H2B*, *PGK*, *CYC*, *TUA*, and *UBCE*, were retrieved from our *Lycium* EST database (Table S2). According to the reference gene sequence, primers were designed using Primer3 (http://frodo.wi.mit.edu/primer3/) based on these criteria: GC% of 40–80%, Tm of 60°C, length of 18–24 bp, and PCR product length of 150–250 bp (See Table 1 for detailed primer sequence information). Gel electrophoresis was performed to confirm PCR product amplification. In addition, PCR products were cloned and sequenced to confirm amplicon correspondence to the reference gene. qRT-PCR was performed in an optical 96-well plate with a LightCycler480 detection system (Roche systems) and universal cycling conditions (30 s 95°C, 40 cycles of 15 s at 95°C and 60 s at 60°C) followed by a dissociation curve to assure specific amplification. For the qRT-PCR reaction, 2 µl 10X SYBR Green Master Buffer (RR047A, Takara, Japan), 10 µM of a gene-specific forward and reverse primer, and 20 µg cDNA template were mixed in each 20 µl reaction. To evaluate PCR efficiency, calibration curves of a cDNA five-fold dilution series were constructed to calculate PCR efficiency and the regression coefficient (R^2^) for each candidate reference gene.

**Table 1:** Primer sequences and amplification efficiencies of the candidate reference genes used in this study.

Genes	Primer Name	Primer Sequence (5′-3′)	Product Length (bp)	PCR Efficiency (%)±SD		R^2^	
				*Lr*	*Lb*	*Lr*	*Lb*
*Actin*	ACTIN1-F	CTCAGCACCTTCCAGCAGAT	162	99.8±1.4	94.8±5.2	1	0.9996
	ACTIN1-R	TAACACTGCAACCGCATTTC					
	ACTIN2-F	CACCTTCCAACAGATGTGGATT	150	98.0±2.5	99.0±8.1	0.9984	0.9956
	ACTIN2-R	TCCTGCTCAGAACTCCGACT					
*EF1α*	EF1α-F	GAAGGGTGTCCCTCAGATCA	180	101.2±1.2	100.7±4.7	0.998	0.9997
	EF1α-R	CCGTCCATGTCGTCTCTTTT					
*GAPDH*	GAPDH1-F	CAGTGGAATTGGATCACAAGG	191	104.1±4.2	103.3±4.5	0.9963	0.9771
	GAPDH1-R	AACTGGTTTCAAGTTCACTACCATC					
	GAPDH2-F	CAGGTGTTGCAGTACCAGGA	192	104.4±1.1	99.8±1.6	0.9939	0.9949
	GAPDH2-R	TGGGACTTCCCACTCAGTTT					
	GAPDH3-F	GGTCATGGGAGATGACATGG	208	109.6±2.2	ND	0.9874	ND
	GAPDH3-R	TCTCGTTACAATTTTCAGAACAGG					
*UBQ*	UBQ-F	TGAATGTGGTGCTGGAACTT	202	105.6±8.7	107.5±6.2	0.9910	0.9513
	UBQ-R	GGTTGCCACATACATCAAAAA					
*SAMDC*	SAMDC1-F	TTCAGTAGCGGTGGTAGCTG	169	102.8±3.4	108.3±1.5	0.9983	0.9831
	SAMDC1-R	CATTATTTGCATGCCTGGAT					
	SAMDC2-F	ATCTCCCACATCCGTTCTGA	200	105.0±3.4	99.9±2.2	0.9982	0.9965
	SAMDC2-R	TCAGAATTTGCACACAGACG					
*H2B*	H2B1-F	AGTGCTTCCTGGTGAATTGG	163	96.3±2.1	87.6±7.7	0.9944	0.9994
	H2B1-R	TGGATAATACCTAGCCCTAGTTTCC					
	H2B2-F	GTTGGTGCTTCCTGGTGAAT	156	102.9±8.1	94.1±9.4	0.9678	0.9996
	H2B2-R	TCAAACAGAAACCCTAAACAGGA					
*PGK*	PGK1-F	GGACTTGCCGAGAAGATGAG	154	105.3±1.4	ND	0.9975	ND
	PGK1-R	CAGAAGGTGCAAAAGGAAAAA					
	PGK2-F	CAGTTTGGAGCTACTGGAAGG	150	100.8±1.1	95.1±1.1	0.9943	0.9983
	PGK2-R	ACTCAATGTTTCCCAACTGGA					
	PGK3-F	ATGCTTCGTGGGACATGTAA	154	96.3±3.9	ND	0.9948	ND
	PGK3-R	AAAAGTGAGCACCCACAAGC					
*CYC*	CYC-F	AATGGCTTGATGGGAAACAT	172	89.1±0.4	ND	0.9969	ND
	CYC-R	TCAACGTTTCATAACATGATCAAC					
*TUA*	TUA1-F	TGAAGCACGTGAAGATCTGG	161	88.1±2.7	ND	0.9971	ND
	TUA1-R	TGCAGCAACTGAAGAACACC					
	TUA2-F	GTTGGTGAGGGCATGGAG	153	104.7±0.9	ND	0.9995	ND
	TUA2-R	TTCAGCAGCCAAATTCATTG					
*UBCE*	UBCE-F	TTCCCAACTTGGTTGTTGCT	157	95.2±7.8	ND	0.9931	ND
	UBCE-R	ACCAGAGCAGGGATGACAAC					

Note: ND, not determined. Lr, *L. ruthenicum*; Lb, *L. barbarum*.

### Verification of Fruit-specific and Stress Responsive Gene Expression

To validate the selected reference gene, relative expression of several genes were measured, including *LrPG* homologous to *SlPG* involved in fruit ripening [7], [8], *Lycium NAC* homologous to the salt-stress responsive gene *SlNAC1* [6], and anthocyanin genes. Expression of structural genes (*F3′H* and *F3′5′H*) and BMW regulatory genes involved in anthocyanin biosynthesis were also investigated. Two sets of primers were designed for both *F3′5′H* and *F3′H* genes and used for qRT-PCR assay. PCR products amplified by one set of primers corresponded to the conserved functional domain of F3′5H or F3′H protein. Thus, this primer pair was used to estimate transcripts of all copies of *LrF3′5′Hs* or *LrF3′Hs*. Another set of gene-specific primers across the coding region and the 3′ untranslated region was designed for *LrF3′5H1* or *LrF3′H1* to measure expression of the single copy of *LrF3′5H1* or *LrF3′H1*. These primers were designed using Primer3 (See Table S3). All experiments were performed in triplicate.

## Results

### Characterization of Potential Reference Genes in *Lycium*

To identify stable reference genes suitable for particular tissues and/or experimental condition (Table S1), we retrieved 18 candidate reference genes from the *Lycium* EST library (Zeng *et al*. unpublished data). Gene function, primer sequence, amplicon size, and PCR efficiency of these potential reference genes are shown in Tables 1 and S2. Amplification specificity for each gene was confirmed by agarose gel electrophoresis (Fig. S2) and single peaks of melting curves (Fig. S3). A calibration curve was created for each gene tested using a serial five-fold dilution. Subsequently, a significant linear relationship between cycle number and dilution-fold was confirmed for calibration curves in both *L. ruthenicum* and *L. barbarum* (Table 1). As shown in Table 1, PCR efficiency for these candidate reference genes ranged from 88.1% for *TUA1* to 105.1% for *PGK1* in *L. ruthenicum* and from 87.6% for *H2B1* to 108.3% for *SAMDC1* in *L. barbarum*.

### Candidate Reference Gene Expression

**Figure 1:**
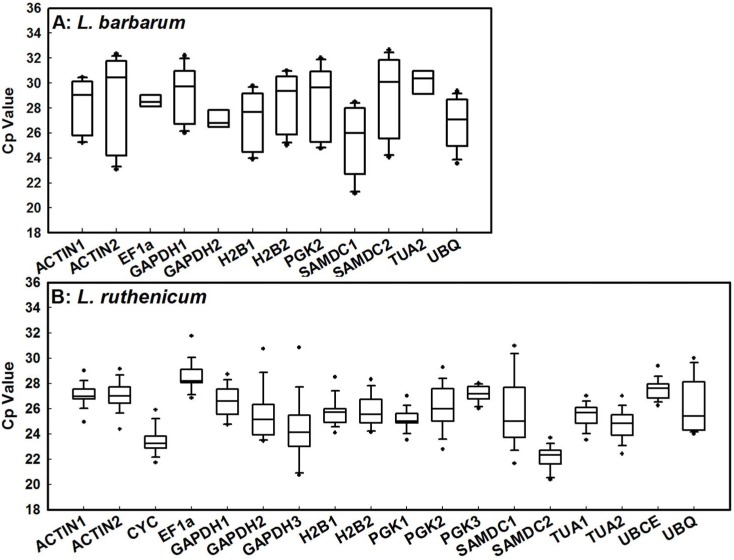
RNA transcription levels of candidate reference genes presented as Cp mean value in different samples of *L. barbarum* (A) and *L. ruthenicum* (B).

To identify stable reference genes, expression of 18 candidate reference genes across all samples were detected by qRT-PCR. The variations in candidate reference gene mRNA were revealed by the spectrum of Cp values across all samples. Theoretically, the candidate reference gene with the least amount of variation is the most stable. As shown in Fig. 1A, *EF1α*, *GAPDH2*, and *TUA2* were more stably expressed across *L. barbarum* samples than remained reference genes. In *L. ruthenicum*, transcripts of all candidate reference genes except *SAMDC1* and *UBQ* were stable across all samples (Fig. 1B). Interestingly, the Cp variance of a candidate reference gene in *L. barbarum* was greater than its counterpart in *L. ruthenicum* (Fig. 1). These results indicate that no candidate reference gene was consistently expressed across different tissues, experimental treatments, or species. Therefore, identifying the best reference gene target is necessary for normalizing gene expression in a particular experimental system.

### Normfinder-determined Rank of Candidate Reference Genes

**Figure 2:**
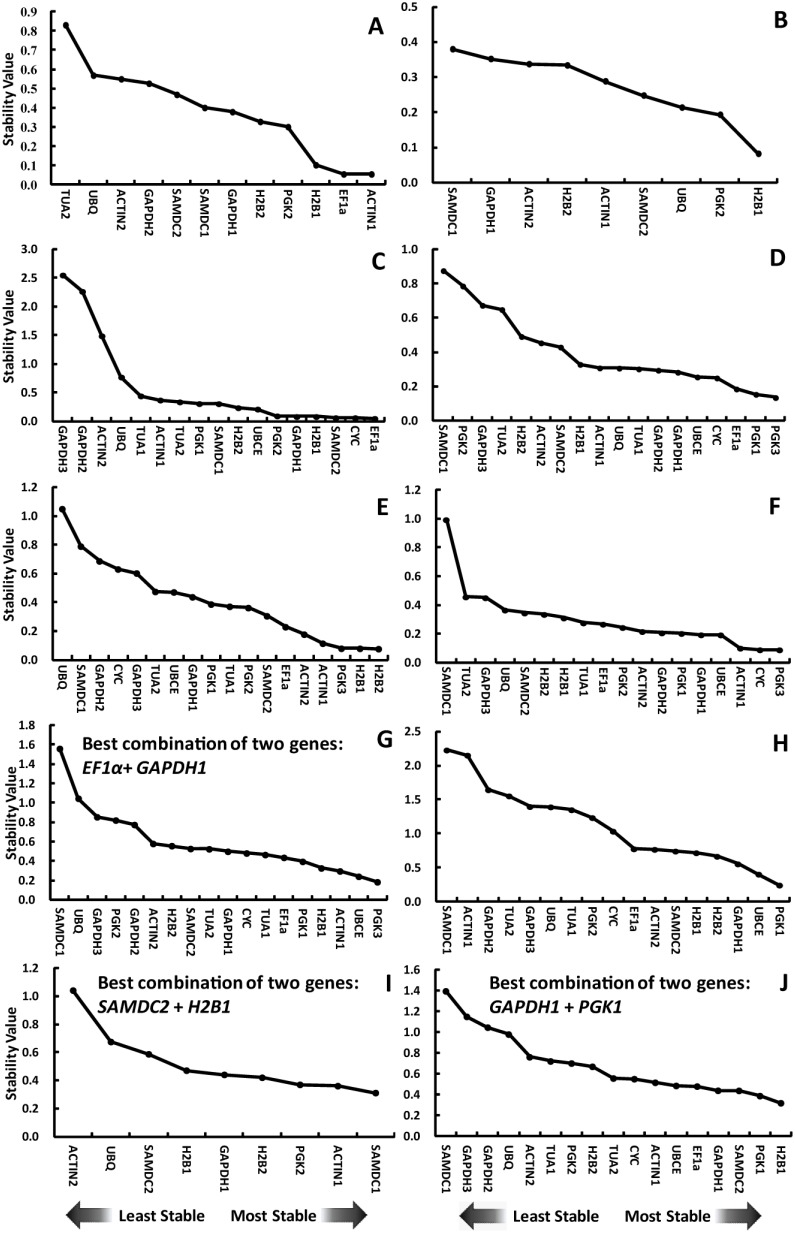
Rank of candidate reference genes by NormFinder procedure based on data generated by qRT-PCR analysis in *L. barbarum* fruits (A), *L. barbarum* roots treated by salt (B), *L. ruthenicum* fruit (C), *L. ruthenicum* leaves treated by salt (D), *L. ruthenicum* roots treated by salt (E), *L. ruthenicum* stems treated by salt (F), *L. ruthenicum* leaves, roots, and stems treated by salt (G), different *L. ruthenicum* tissues (H), all *L. barbarum* samples (I), and all *L. ruthenicum* samples (J).

For NormFinder analysis, inter- and intra-group variations were taken into account, and both results were combined and presented as the stability value of each candidate reference gene. Candidate reference genes with lower stability values are more stably expressed. In *L. barbarum* fruits, the most stable reference gene was *ACTIN1* followed by *EF1α* and *H2B1* (Fig. 2A). The reference gene target was *H2B1*, which was suitable for salt-treated *L. barbarum* roots (Fig. 2B). When evaluating across *L. barbarum* samples, the top two ranked stable reference genes were *SAMDC1* and *ACTIN1* and the best combination of reference genes were *SAMDC2*+*H2B1* (hereafter reference gene A+B corresponding to the best stable reference gene A combined with B; Fig. 2I). The stability value for *SAMDC2*+*H2B1* is 0.099, which is lower than that of *SAMDC1* (0.310). In *L. ruthenicum* fruit, *EF1α* and *CYC* were the top two ranked stable reference genes followed by *SAMDC2*, *H2B1*, *GAPDH1*, and *PGK2* (Fig. 2C). *PGK3*, *PGK1*, *EF1α*, *CYC* were the most four stable reference genes in salt-treated *L. ruthenicum* leaves (Fig. 2D). In salt-treated *L. ruthenicum* roots, the stability values of *H2B2*, *H2B1*, *PGK3*, and *ACTIN1* were lower than that of remaining candidate reference genes (Fig. 2E). *PGK3*, *CYC*, *ACTIN1*, and *UBCE* were more stably expressed than other candidate reference genes in salt-treated *L. ruthenicum* stems (Fig. 2F). The top four ranked candidate reference genes with *PGK3*, *UBCE*, *ACTIN1*, and *H2B1* in all salt-treated *L. ruthenicum* samples were imperfectly identical to that in leaves, roots, and stems (Figs. 2D–2G). Furthermore, the best combination of two genes optimal to evaluate the expression of genes in salt-treated *L. ruthenicum* seedlings were *EF1α*+*GAPDH1*, the stability value of which was 0.112 lower than that of the best single reference gene *PGK3* (0.185). Correctly estimating the spatial expression profile of genes in *L. ruthenicum* is essential to elucidate their biological function. The top four ranked most stable reference genes were *PGK1*, *UBCE*, *GAPDH1*, and *H2B2* across stems, leaves, roots, sepals, petals, stamens, and pistils (Fig. 2H). When all samples were analyzed together, the best single stable reference gene was *H2B1* and the best combination of two genes were *GAPDH1*+*PGK1* (Fig. 2J). The stability value of *GAPDH1*+*PGK1* was 0.182 lower than that of *H2B1* (0.321). Noticeably, *SAMDC1* was the most unstable reference gene in salt-treated samples and among different tissues of *L. ruthenicum*, which corresponded with its large Cp variance (Figs. 1 and 2).

### geNorm-determined Rank of Candidate Reference Genes

**Figure 3:**
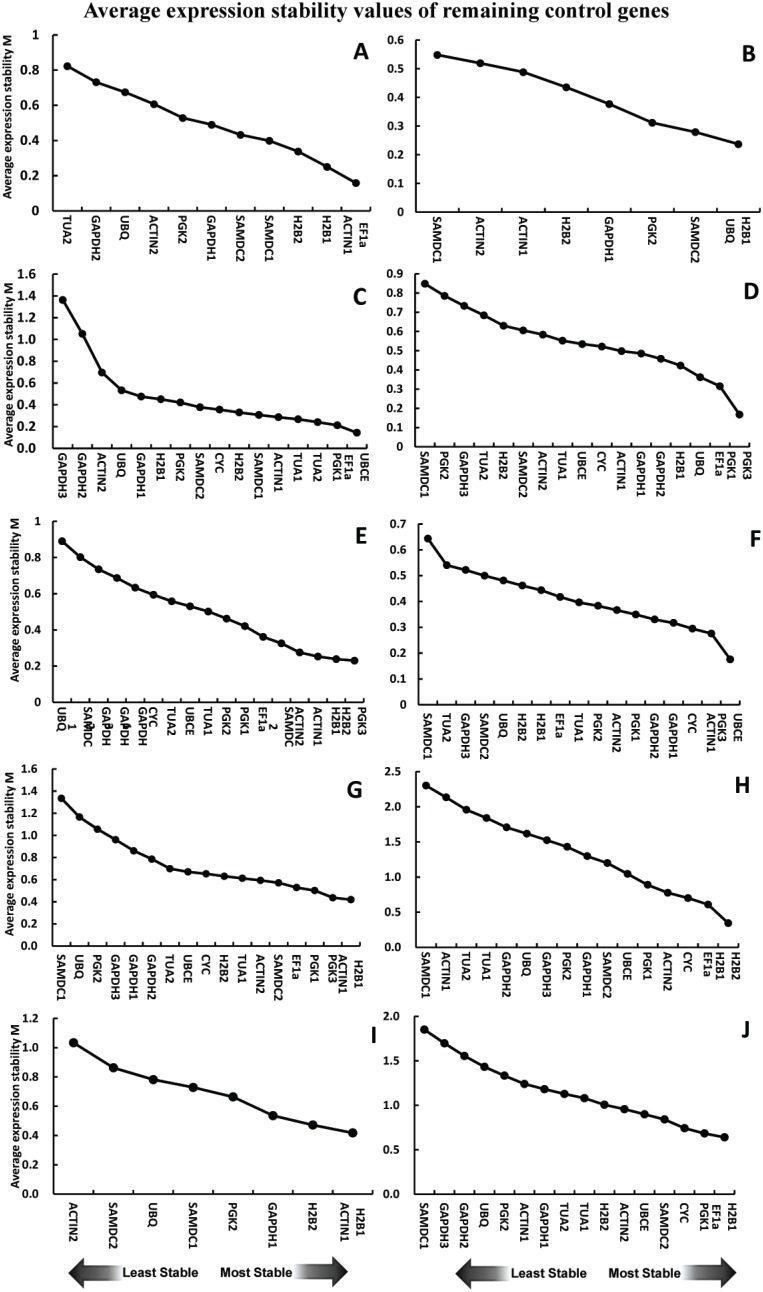
Rank of candidate reference genes by geNorm procedure based on data generated by qRT-PCR analysis in *L. barbarum* fruits (A), *L. barbarum* roots treated by salt (B), *L. ruthenicum* fruit (C), *L. ruthenicum* leaves treated by salt (D), *L. ruthenicum* roots treated by salt (E), *L. ruthenicum* stems treated by salt (F), *L. ruthenicum* leaves, roots, and stems treated by salt (G), different *L. ruthenicum* tissues (H), all *L. barbarum* samples (I), and all *L. ruthenicum* samples (J).

geNorm offers the average expression stability measure (M) of a gene as the average pair-wise variation of a particular gene compared to the remaining candidate reference genes. The more low M values indicated the more stability of candidate reference gene. As shown in Fig. 3A, *EF1α*/*ACTIN1* (hereafter reference gene A/B geNorm-designated as top 1 stable reference gene A and/or B) with the lowest M values was the most stable reference genes in *L. barbarum* fruits. In salt-treated *L. barbarum* roots, *H2B1*/*UBQ* was the best choice of reference genes (Fig. 3B). *H2B1/ACTIN1* was the most stable reference genes when evaluating across fruits and roots samples of *L. barbarum* (Fig. 3I). In *L. ruthenicum* fruit samples, *UBCE*/*EF1α* was the most stable reference genes (Fig. 3C). As shown in Figs. 3D–3F, *PGK3*/*PGK1*, *PGK3*/*H2B2*, and *UBCE*/*PGK3* were with the lowest M value in salt-treated *L. ruthenicum* leaves, roots, and stems, respectively. Also, the most stable reference gene was *H2B1*/*ACTIN1* when all salt-treated *L. ruthenicum* samples analyzed (Fig. 3G). In addition, *H2B2*/*H2B1* was the best reference gene stably expressed across *L. ruthenicum* stems, leaves, roots, sepals, petals, stamens, and pistils samples (Fig. 3H). When all *L. ruthenicum* samples were evaluated, *H2B1*/*EF1α* was the most stable reference gene (Fig. 3J).

**Figure 4:**
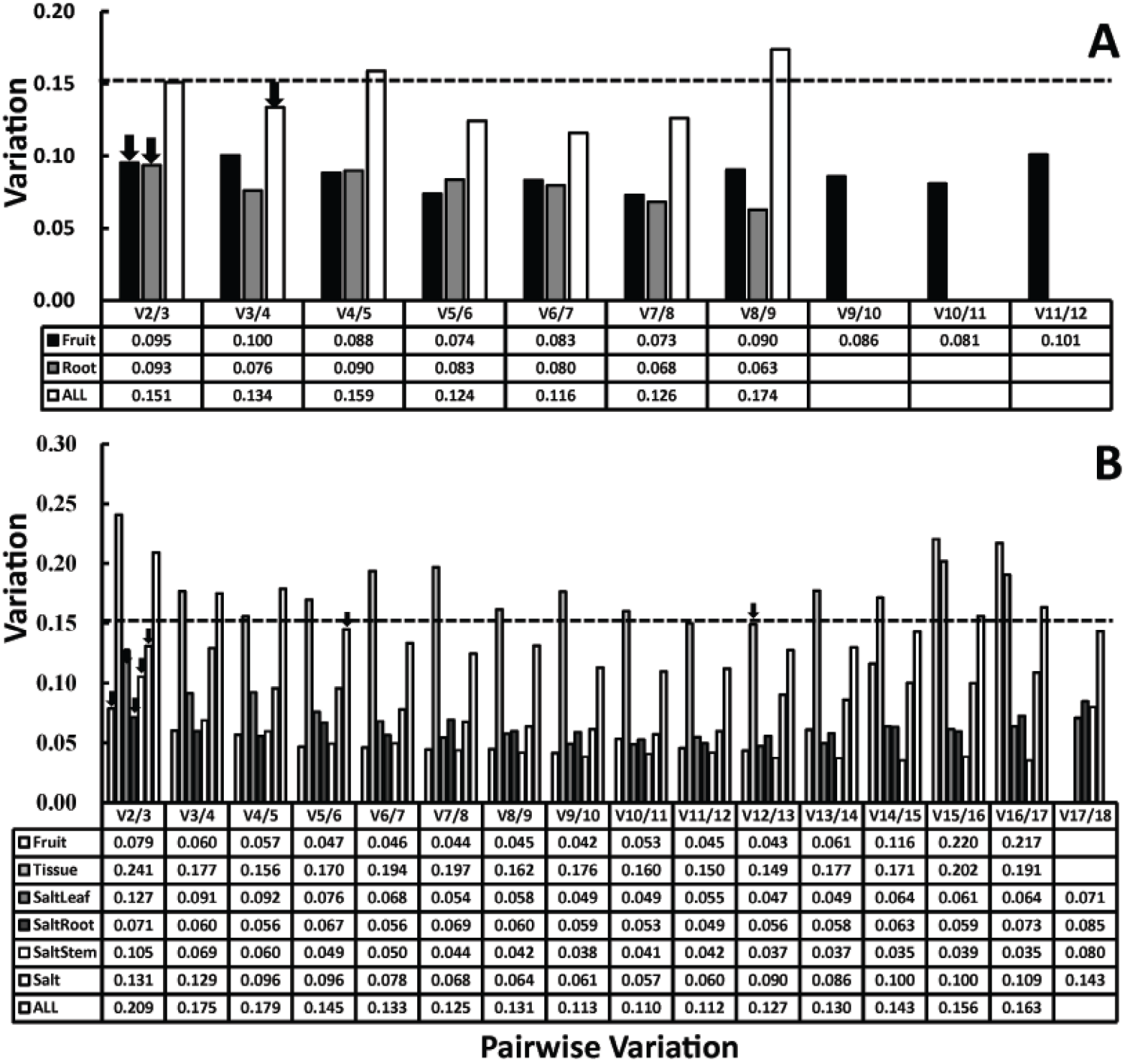
Pairwise variation analysis of the candidate reference genes in samples of *L. barbarum* (A) and *L. ruthenicum* (B) by geNorm procedure. The pairwise variation (V_n_/V_n+1_) was calculated between the normalization factors NFn and NFn+1 by the geNorm software to determine the optimal number of reference genes required for qRT-PCR data normalization. The black arrow indicated the optimal number of reference genes.

Generally, gene expression normalized by one stable reference gene is insufficient for reliable and precise results. The optimal number of reference genes required for reliable normalization was statistically predicted by geNorm with a V value (cut-off = 0.15), representing pairwise variation. In our work, V value analysis indicated that the ideal number of reference genes in single tissue was two, and that more than two reference genes were required to correctly normalize gene expression in more than two tissues (Fig. 4). For instance, combining three reference genes, *H2B1*+*H2B2*+*ACTIN1*, were enough to simultaneously and accurately normalize gene expression in both fruits and roots of *L. barbarum* (Fig. 4A). One exception was apparent: two reference genes (*H2B1*+*ACTIN1* with V2/3 value of 0.131<0.15) were enough to synchronously evaluate gene expression in salt-treated *L. ruthenicum* stems, leaves, and roots (Fig. 4B). At least five reference genes (*H2B1*, *EF1α*, *PGK1*, *CYC*, *SAMDC1*) were required to precisely normalize gene expression across all *L. ruthenicum* samples (Fig. 4B).

### Validation of Target of Reference Genes

To confirm whether normalization by different candidate reference genes altered qRT-PCR-measured expression of genes of interest, expression of several genes related to salt stress response, anthocyanin biosynthesis, and fruit ripening were analyzed.

**Figure 5:**
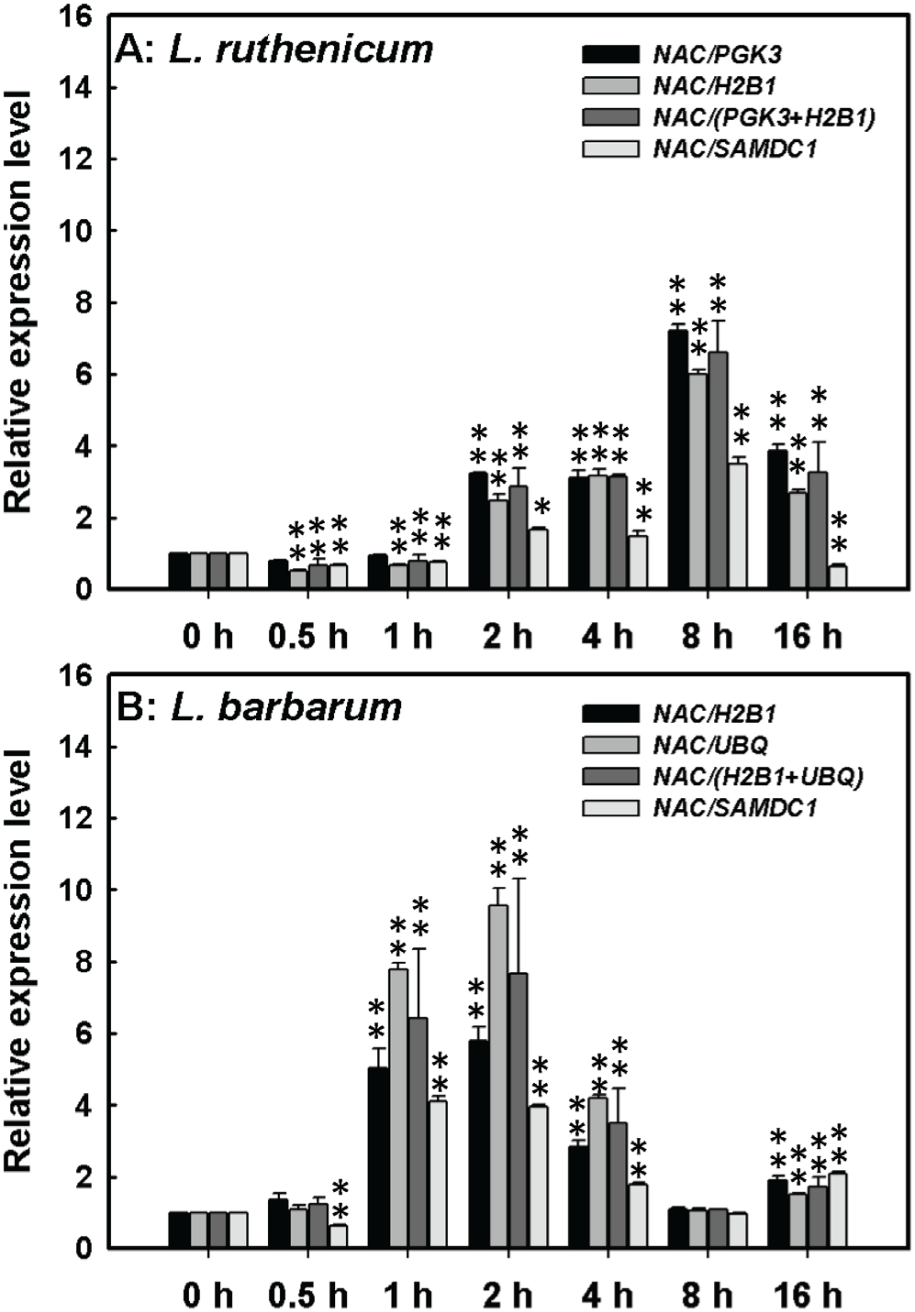
Relative quantification of *Lycium NAC* gene using validated reference genes for normalization in salt-treated *L. ruthenicum* (A) and *L. barbarum* (B) roots. The validated reference genes used as normalization factor were *PGK3* and *H2B1* for *L. ruthenicum* and *H2B1* and *UBQ* for *L. barbarum*, which were most stable reference genes recommended by geNorm and/or NormFinder procedure(s). The most unstable gene *SAMDC1* was also selected as a comparative normalization factor to evaluate *NAC* gene expression in both *Lycium* species. Double asterisks show statistical significant differences between samples salt-treated and control sample at *P*<0.01 level.

For salt stress, a *Lycium* NAC transcription factor was selected to validate its expression level in salt-treated *Lycium* roots. Amino acid sequence analysis revealed that *Lycium* NAC was 85% identical to *Solanum lycopersicum* NAC (SlNAC, NP_001234482), which was previously identified as a salt stress-responsive genes in tomato roots [6]. In *L. ruthenicum* roots, the top two ranked reference genes *PGK3* and *H2B1*, recommended by geNorm and/or NormFinder (Fig. 2E and 3E), were chosen to evaluate *Lycium NAC* expression (Fig. 5A). *SAMDC1*, serving as an unstable reference gene, was also used to comparatively assess *NAC* expression (Fig. 5A). As shown in Fig. 5A, *LrNAC* expression revealed by qRT-PCR using reference genes *PGK3*, *H2B1*, *PGK3*+*H2B1*, and *SAMDC1* were similar. However, the fold-change of *LrNAC* expression at 8 h compared to 16 h was 1.89 for *PGK3*, 2.24 for *H2B1*, and 2.02 for *PGK3/H2B1*, which were lower than that of 5.65 for *SAMDC1* (*P*<0.01; Fig. 5A). These results indicate that exact expression of *NAC* as revealed by stable reference genes *PGK3* and/or *H2B1* were more precise than those calculated using the unstable gene *SAMDC1*. In *L. barbarum* roots, *H2B1* and *UBQ*, identified by NormFinder and/or geNorm as the best stable reference genes, were utilized as internal controls to estimate *LbNAC* expression (Fig. 5B). Also, *LbNAC* expression was estimated with the unstable reference gene *SAMDC1* as an internal control. As presented in Fig. 5B, the qRT-PCR-determined expression of *LbNAC* using stable reference genes *H2B1*, *UBQ*, or *H2B1+UBQ* as internal control were in agreement, which is a different finding from those revealed by the unstable reference gene *SAMDC1*. Summarily, in salt-stress treated *Lycium* seedling roots, more precise and reliable results were offered by stable reference genes systematically identified by geNorm and NormFinder compared to unstable reference gene (Fig. 5).

**Figure 6:**
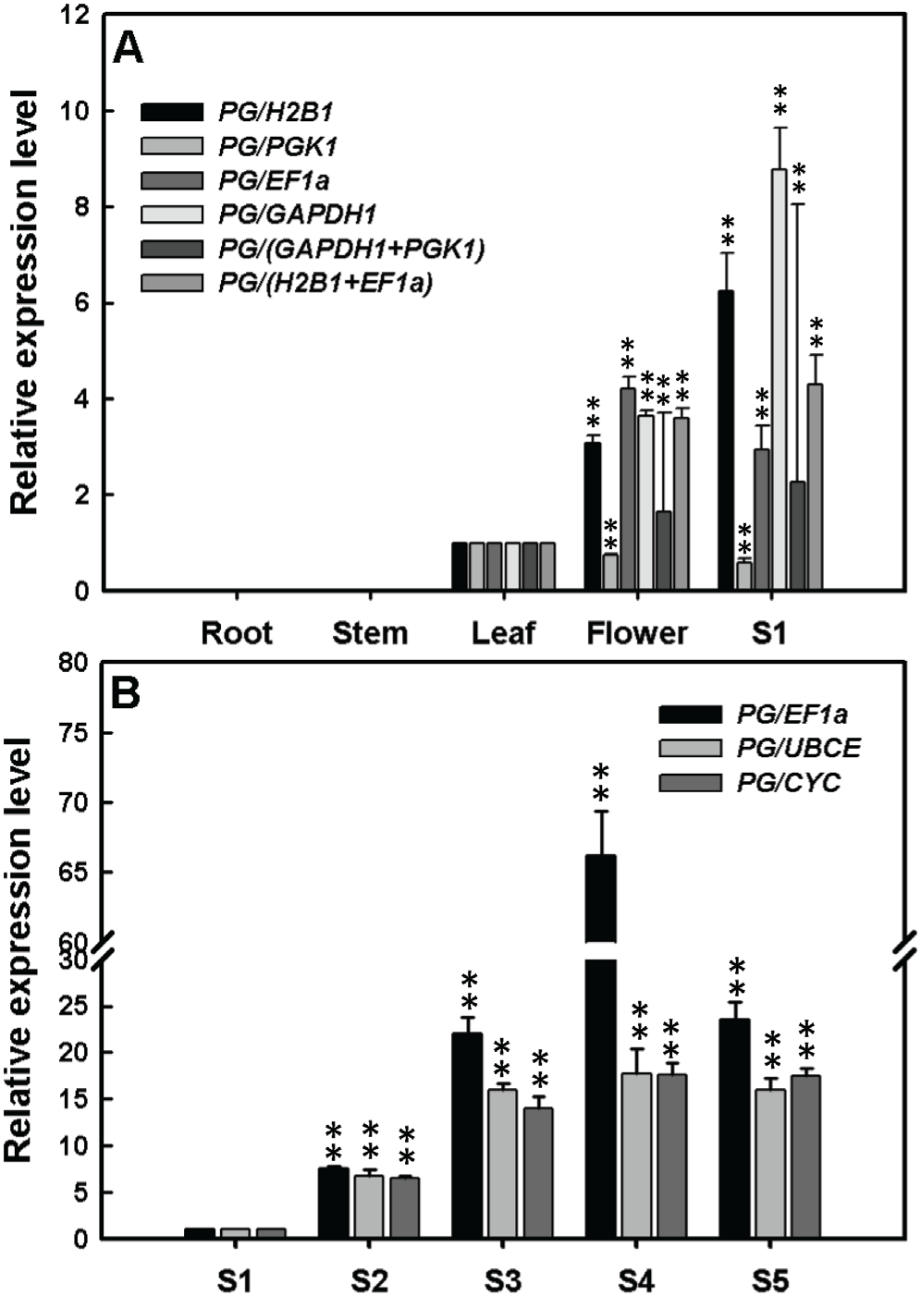
Relative quantification of a putative fruit-specific expression gene *LrPG* in different tissues (A) and developmental fruits (B) of *L. ruthenicum*. **A**, the validated reference gene(s) were *H2B1*, *PGK1*, *EF1α*, and *GAPDH1*. Of those, *H2B1* and *H2B1*/*EF1α* were the most stable reference genes recommended by NormFinder and geNorm, respectively. The stability value of the best combination genes *GAPDH1*+*PGK1* recommended by NormFinder was lower than that of the best single reference gene *H2B1*. Double asterisks show statistical significant differences between flowers or S1 fruits and leaves at *P*<0.01 level. **B**, the top2-ranked most stable reference genes *EF1α* and *CYC* NormFinder-determined and *UBCE* and *EF1α* geNorm-identified were used as normalization factors to evaluate *LrPG* expression in ripening fruits. Double asterisks show statistical significant differences between S2–S5 fruits and S1 fruits at *P*<0.01 level.

To identify candidate fruit-specific expression genes in *L. ruthenicum*, an EST sequence encoding *PG* homologous to tomato *PG*, which was previously reported to be fruit-specific expression [8], [9], [31], was retrieved from a *Lycium* EST library. Expression of *LrPG* in roots, stems, leaves, flowers, and ripening fruits was investigated using several reference genes recommended by NormFinder and/or geNorm (Fig. 6). With regard to the selection of reference genes, *H2B1*/*EF1α* and *H2B1* and *PGK1* were recommended as the most stable top two ranked reference genes across all samples by geNorm and NormFinder, respectively (Fig. 2J and 3J). Additionally, NormFinder predicted that the stability value of the best combination of two genes *GAPDH1*+*PGK1* was lower than that of the single most stable gene *H2B1*, suggesting that *GAPDH1+PGK1* was more stable than *H2B1*. Consequently, these candidate reference genes were selected to evaluate the expression of *LrPG* in roots, stems, leaves, flowers, and stage S1 fruits (Fig. 6A). As shown in Fig. 6A, *LrPG* transcripts were undetectable in roots and stems and were highly expressed in stage S1 fruits followed by flowers and leaves when using the reference genes *H2B1*, *GAPDH1*, *H2B1*+*EF1α*, and *GAPDH1*+*PGK1* as normalization factors. In ripening fruits, expression of *LrPG* was normalized by *EF1α*, *UBCE*, and *CYC*, which were the most stable top two ranked reference genes (Figs. 2C and 3C). Normalized expression of *LrPG* was identical using the three reference genes. Furthermore, *LrPG* transcripts were increasingly enhanced and peaked at stage S4 (Fig. 6B). Summarily, *LrPG* was abundantly expressed in fruits, especially at stage S4 prior to the expansion stage S5 (Fig. 6), suggesting that *LrPG* was involved in *L. ruthenicum* fruit ripening.

**Figure 7:**
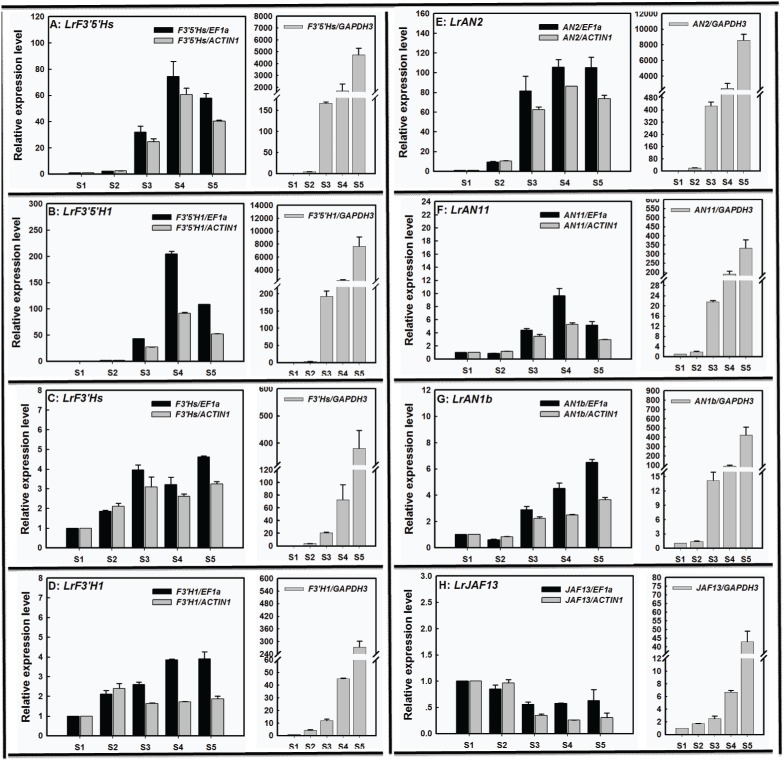
Relative quantification of structural and regulatory genes involved in anthocyanin biosynthesis in *L. ruthenicum* fruits using validated reference genes for normalization. The validated *EF1α*, *ACTIN1*, and *GAPDH3*, respectively representing the most stable, medium stable, and unstable reference genes recommended by both geNorm and NormFinder, were used as normalization factor.

For anthocyanin biosynthesis in *L. ruthenicum* fruits, several genes, including structural genes (*F3′5′H* and *F3′H*) and regulatory genes homologous to petunia *AN2*, *AN11*, *JAF13*, and *AN1*, were used as target genes of interest to demonstrate the utility of the validated target reference genes in qRT-PCR (Fig. 7). To better understand underlying molecular mechanism of most metabolites being introduced to the delphindin branch in *L. ruthenicum* fruits, we measured expression of *LrF3′5′H* and *LrF3′H* during fruit development. Expression level of these genes was normalized using the most stable reference gene, *EF1α*, a moderately stable reference gene, *ACTIN1*, and an unstable reference gene, *GAPDH3*. With *EF1α* as an internal control, expression profiles of these genes were consistent with those normalized by the moderately stable reference gene *ACTIN1* (Fig. 7). However, gene expression normalized with the unstable reference gene *(GAPDH3*) was significantly different from those estimated with *EF1α* or *ACTIN1* (Fig. 7). Furthermore, gene transcripts were over-estimated at stage S4 and/or S5 when *GAPDH3* was used as an internal control. These results suggest that using an unstable reference gene(s) may give rise to less accurate or misleading results.

## Discussion

*L. barbarum* and *L. ruthenicum* are widely utilized as traditional Chinese medicine (TCM) because of their purported health-promoting phytochemical compounds [1], [2], [32], [33]. Such phytochemicals include carotenoids and anthocyanins and which are thought to have anti-aging, neuroprotective [12], anti-diabetic, anti-glaucoma, anti-oxidant, immunomodulatory, anti-tumor and cytoprotective effects. To date, the biosynthetic and regulatory mechanisms of these phytochemicals are unclear and how *Lycium* resists abiotic stresses (saline and alkaline conditions) remains uncertain. Both *L. barbarum* and *L. ruthenicum* plants are tolerant to salt and alkalinity, so precise evaluation of gene expression is needed to identify the functional genes involved in physiological and/or biological processes that mediate these stress-resistant properties.

qRT-PCR has been used to quantify gene expression [17], and reliable qRT-PCR results depend on the choice of a stable reference gene(s). However, increasing evidence indicates that HKG expression, which is used as a candidate reference gene, is variable across different tissue samples and/or experimental conditions [24], [25]. Thus, systematically identifying stable reference genes prior to their use in qRT-PCR normalization is necessary [27], [28]. Because few reference genes have been systematically evaluated in *L. barbarum* ripening fruits [18], we identified suitable reference genes in *Lycium* samples from different tissues and in plants grown under salt stress.

First, we measured expression stability of 18 candidate reference genes across seven experimental sets (Table S1). NormFinder and geNorm procedures were used to calculate and identify the best suitable reference genes for specific investigations (Figs. 2 and 3). Generally, both geNorm and NormFinder identify the same subset of reference genes but rank them differently in certain tissues or under stresses (Figs. 2 and 3). For instance, although geNorm and NormFinder identified *ACTIN1*, *EF1α*, and *H2B1* in *L. barbarum* fruits as the most stable reference genes, their ranks were different in each algorithm (Figs. 2A and 3A). Similarly, *PGK3*, *CYC*, *ACTIN1*, and *UBCE* which differed with respect to rank were identified as the most stable reference genes in salt-treated *L. ruthenicum* stems with these two procedures (Figs. 2F and 3F). This situation agree with a previous study of *L. barbarum* fruits [18]. However, in citrus case, remarkable divergence was observed in the most stable reference genes ranked by geNorm and NormFinder [34]. Divergence in the top-ranked stable reference genes predicted by the two procedures could be attributed to differences in statistics used by geNorm and NormFinder.

Previous research indicates that *GAPDH*/*EF1α* is the best stable reference gene for normalizing gene expression in *L. barbarum* ripening fruits [18]. Noticeably, *GAPDH* and *EF1α* were in a previous *L. barbarum* study [18] and have high and low sequence identity with *L. ruthenicum GAPDH3* and *EF1α*, respectively. However, *GAPDH3* was identified as the unstable reference gene in *L. ruthenicum* fruits (Figs. 2C and 3C). Differences in expression stability between *L. ruthenicum GAPDH3* and *L. barbarum GAPDH* may be species-specific. Alternatively, the set of candidate reference genes investigated possibly affect the rank of reference genes. In our study, the best stable reference genes in *L. barbarum* fruits were *EF1α* and *EF1α*/*ACTIN1* as predicted by NormFinder and geNorm, respectively (Figs. 2A and 3A). Also, *EF1α* and *EF1α*/*UBCE* were respectively recommended by NormFinder and geNorm as the most stable reference gene(s) in *L. ruthenicum* ripening fruits (Figs. 2C and 3C). Obviously, the best stable reference gene in *Lycium* ripening fruits was *EF1α*, which is often used in various other species [17], [35]–[37].

In addition, different tissue samples had unique stable reference genes among the 18 studied (Figs. 2D–2F and 3D–3F). NormFinder analysis of salt-treated *L. ruthenicum* leaves indicated that the top five ranked most stable reference genes were *PGK3*, *PGK1*, *EF1α*, *CYC*, and *UBCE*, which was different from *H2B2*, *H2B1*, *PGK3*, *ACTIN1*, and *ACTIN2* for salt-treated roots and *PGK3*, *CYC*, *ACTIN1*, *UBCE*, and *GAPDH1* for salt-treated stems (Figs. 2D–2F), which was confirmed by geNorm-determined results (Figs. 3D–3F). Previous studies also document alterations in candidate reference gene rankings in distinct sample sets [14], [19], [38], [39].

To validate the geNorm- and NormFinder-determined results for suitable reference genes in experimental systems, expression of several genes was investigated (Figs. 5–7). Previous study demonstrated that the salt-sensitive gene *SlNAC1* is significantly upregulated in tomato roots [6]. As presented in Fig. 5, *LrNAC* and *LbNAC*, *SlNAC1* homologs, were significantly upregulated by salt stress. Specifically, *LrNAC* transcripts decreased in the first 1 h followed by a significant increase at 2 h and peaked at 8 h whereas *LbNAC* transcripts were enhanced at 1 h and almost peaked at 2 h. The divergent expression pattern of *Lycium NACs* suggested that cis- and/or trans-elements controlling *NAC* expression responding to salt stress were evolutionarily divergent in *Lycium* species. In this case, using the unstable reference gene *SAMDC1* as an internal control offered less precise expression patterns for *LrNAC* and incorrect expression profiles for *LbNAC* (Fig. 5).

In tomato, several genes, including *PG* [8], [9], [31] and *E8* [40], [41], were reported to be relative to fruit ripening. In this study, a unigene encoding *LrPG* was retrieved from our EST database (Zeng *et al.,* unpublished data) and data show that *LrPG* was highly expressed in stage S1 fruit when using the best single reference gene *H2B1*, or the best combination of two genes *GAPDH1*+*PGK1* and *H2B1*+*EF1α* as an internal control (Fig. 6A). During *L. ruthenicum* fruit ripening, *LrPG* transcripts were enhanced and peaked at stage S4 (Fig. 6B). These results suggested that *LrPG* expression was fruit-specific, and that the *LrPG* promoter would be a good choice for investigating the function of genes of interest in *L. ruthenicum* fruit.

Petunidins are abundantly accumulated in *L. ruthenicum* fruits [1], so expression of structural and regulatory genes involved in anthocyanin biosynthesis was normalized using the most stable, moderately stable, and most unstable reference genes (Fig. 7). As shown in Fig. 7, expression of genes normalized by the unstable reference gene *GAPDH3* was significantly distinct compared to those normalized by the most stable reference gene *EF1α* or the moderately stable reference gene *ACTIN1*, suggesting that normalization using unstable reference gene resulted in misinterpretation of anthocyanin gene expression. These results also indicated that the moderately stable reference gene *ACTIN1* was stable enough to precisely calculate the expression of anthocyanin genes in ripening fruit. qRT-PCR data suggested that, except for *LrJAF13*, all tested gene expressions were enhanced at the first four stages when using *EF1α* or *ACTIN1* as a normalization factor (Fig. 7). Furthermore, the fold-change of *LrF3′5′H* transcripts at the first four stages was higher than that of *LrF3′H* when using internal gene *EF1α* or *ACTIN1* (Figs. 4A–4D), and these data were confirmed with data generated with *LrF3′H* as an internal control (Fig. S4). These results may account for 95% of anthocyanins being petunidin-derivatives in mature fruits of *L. ruthenicum* (Figs. 7 and 8, [1]).

## Conclusion

In conclusion, reference gene targets in different tissues and samples under salt-stress were identified in *Lycium*. Data show that reference gene ranking as determined by geNorm and NormFinder were similar with minor change. Our results also indicate that *EF1α* and *ACTIN1* were the top two most stable reference genes in *L. barbarum* fruits, and that *EF1α* was the most stable reference gene in *L. ruthenicum* fruits. Additionally, *H2B1* and *H2B2* were the best reference genes in salt-treated *L. barbarum* and *L. ruthenicum* roots, respectively. Expression of *Lycium NAC*, *LrPG*, and genes involved in *L. ruthenicum* anthocyanin biosynthesis were analyzed to emphasize the importance of validating reference genes to obtain accurate and reliable qRT-PCR results. Results show that *LrPG* expression was fruit-specific, and that *Lycium NAC*s were upregulated and divergent in expression in response to salt stress. Also, both enhanced anthocyanin gene transcripts and increased ratios of *LrF3′5′H*/*LrF3′H* transcript in ripening fruits may have accounted for accumulation of petunidin-derivatives in *L. ruthenicum* fruits. Summarily, reference gene targets identified herein will provide a foundation for achieving accurate and reliable qRT-PCR results and help us understand complex molecular mechanisms of *Lycium* physiological and biological processes such as salt resistance, fruit ripening, and secondary biosynthesis pathways.

## Supporting Information

Figure S1 **A predicted anthocyanin biosynthetic pathway in** ***L. ruthenicum*** **.** According to previous result (Zheng et al. 2011), the petunidin-derivatives are the major component of anthocyanins in *L. ruthenicum* fruits and the anthocyanin pathway was postulated. The arrow weight indicates the size of metabolic flux. The dashed arrows indicated that BMW tricomplex possibly regulate the transcription of *F3′H* and *F3′5′H* gene. CHS, chalcone synthase, CHI, chalcone isomerase; F3′H, flavanone 3-hydroxylase; F3′H, flavonoid 3′hydroxylase; F3′5′H, flavonoid 3′5′hydroxylase; DFR, dihydroflavonol 4-reductase; ANS, anthocyanidin synthase; MT, anthocyanin methyltransferase.
(DOC)

Figure S2 **Electrophoresis analysis of the specificity of primer pairs for RT-PCR amplification.** Of 2.0% agarose gel electrophoresis indicated amplication of a specific product of the expected size for each candidate reference genes. 1, *ACTIN1*; 2, *ACTIN2*; 3, *EF1α*; 4, *GAPDH1*; 5, *GAPDH2*; 6, *GAPDH3*; 7, *UBQ*; 8, *SAMDC1*; 9, *SAMDC2*; 10, *H2B1*; 11, *H2B2*; 12, *PKG1*; 13, *PKG2*; 14, *PKG3*; 15, *CYC*; 16, *TUA1*; 17, *TUA2*; 18, *UBCE*; M, DL2000 DNA Marker.
(DOC)

Figure S3 **Dissociation curves for the eighteen reference genes tested in this study.**
(DOC)

Figure S4 **The expression level of** ***F3*** *′* ***5*** *′* ***H*** **relative to** ***F3*** *′* ***H*** **in** ***L. ruthenicum*** **fruits.** The expression ratio of *F3′5′Hs/F3′Hs* was quantitatively evaluated by qRT-PCR using primers designed on the basis of functionally conserved domains in F3′5′H or F3′H protein while the expression ratio of *F3′5′H1/F3′H1* was quantitatively estimated by qRT-PCR using primers designed across 3′ untranslated region and coding region of *F3′5′H1* or *F3′H1*.
(DOC)

Table S1 **Experimental design to identify reference genes in** ***Lycium*** **species.** Note: TRG, Target of Reference Genes, *indicated that the number of biological replicates.
(DOC)

Table S2 **Annotation of** ***Lycium*** **HKGs.**
(DOC)

Table S3 **qRT-PCR primer for genes used to validate target of reference genes.**
(DOC)
