# Supplementary material for: Identification and Validation of Reference Genes for Quantitative Real-Time PCR Normalization and Its Applications in Lycium
Source: PLoS One. 2014 May 8;9(5):e97039. doi: 10.1371/journal.pone.0097039 (PMC4014596; doi:10.1371/journal.pone.0097039)
Supplement: Figure S1 — A predicted anthocyanin biosynthetic pathway in L. ruthenicum . According to previous result (Zheng et al. 2011), the petunidin-derivatives are the major component of anthocyanins in L. ruthenicum fruits and the anthocyanin pathway was postulated. The arrow weight indicates the size of metabolic flux. The dashed arrows indicated that BMW tricomplex possibly regulate the transcription of F3′H and F3′5′H gene. CHS, chalcone synthase, CHI, chalcone isomerase; F3′H, flavanone 3-hydroxylase; F3′H, flavonoid 3′hydroxylase; F3′5′H, flavonoid 3′5′hydroxylase; DFR, dihydroflavonol 4-reductase; ANS, anthocyanidin synthase; MT, anthocyanin methyltransferase. (DOC) [file pone.0097039.s001.doc]

**
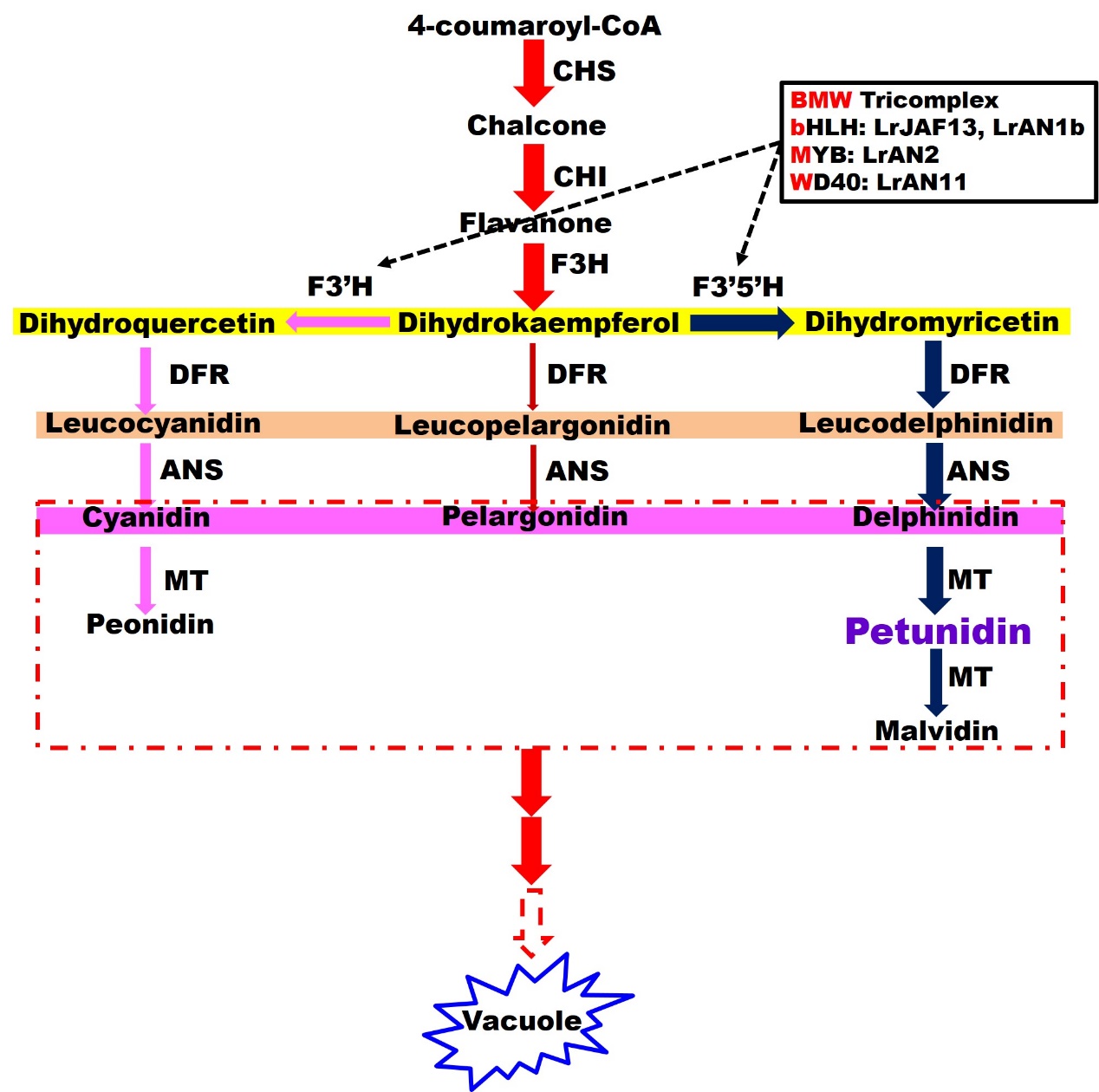
**

**Figure S1 A predicted anthocyanin biosynthetic pathway in *L. ruthenicum*.** According to previous result (Zheng et al. 2011), the petunidin-derivatives are the major component of anthocyanins in *L. ruthenicum* fruits and the anthocyanin pathway was postulated. The arrow weight indicates the size of metabolic flux. The dashed arrows indicated that BMW tricomplex possibly regulate the transcription of *F3’H* and *F3’5’H* gene. CHS, chalcone synthase, CHI, chalcone isomerase; F3H, flavanone 3-hydroxylase; F3’H, flavonoid 3’hydroxylase; F3’5’H, flavonoid 3’5’hydroxylase; DFR, dihydroflavonol 4-reductase; ANS, anthocyanidin synthase; MT, anthocyanin methyltransferase.
