# Supplementary material for: Identification and Validation of Reference Genes for Quantitative Real-Time PCR Normalization and Its Applications in Lycium
Source: PLoS One. 2014 May 8;9(5):e97039. doi: 10.1371/journal.pone.0097039 (PMC4014596; doi:10.1371/journal.pone.0097039)
Supplement: Figure S2 — Electrophoresis analysis of the specificity of primer pairs for RT-PCR amplification. Of 2.0% agarose gel electrophoresis indicated amplication of a specific product of the expected size for each candidate reference genes. 1, ACTIN1; 2, ACTIN2; 3, EF1α; 4, GAPDH1; 5, GAPDH2; 6, GAPDH3; 7, UBQ; 8, SAMDC1; 9, SAMDC2; 10, H2B1; 11, H2B2; 12, PKG1; 13, PKG2; 14, PKG3; 15, CYC; 16, TUA1; 17, TUA2; 18, UBCE; M, DL2000 DNA Marker. (DOC) [file pone.0097039.s002.doc]

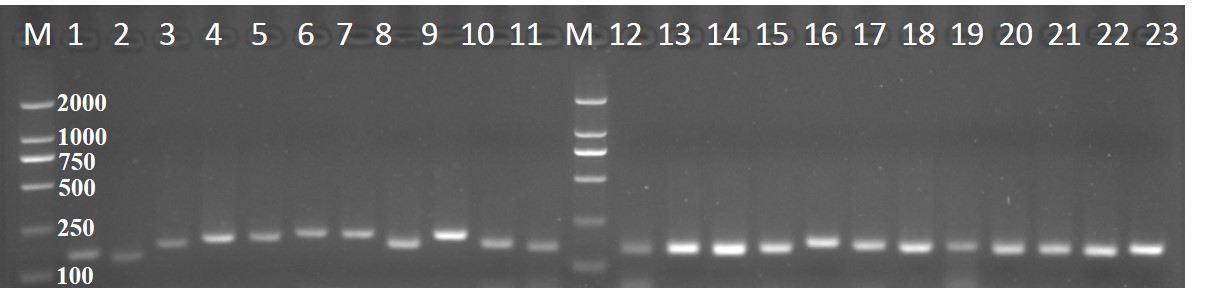


**Figure S2 Electrophoresis analysis of the specificity of primer pairs for RT-PCR amplification.** Of 2.0% agarose gel electrophoresis indicated amplication of a specific product of the expected size for each candidate reference genes. 1, *ACTIN1*; 2, *ACTIN2*; 3, *EF1α*; 4, *GAPDH1*; 5, *GAPDH2*; 6, *GAPDH3*; 7, *UBQ*; 8, *SAMDC1*; 9, *SAMDC2*; 10, *H2B1*; 11, *H2B2*; 12, *PKG1*; 13, *PKG2*; 14, *PKG3*; 15, *CYC*; 16, *TUA1*; 17, *TUA2*; 18, *UBCE*; M, DL2000 DNA Marker
