# Supplementary figures and images for: Identification and Validation of Reference Genes for Quantitative Real-Time PCR Normalization and Its Applications in Lycium
Source: PLoS One. 2014 May 8;9(5):e97039. doi: 10.1371/journal.pone.0097039 (PMC4014596; doi:10.1371/journal.pone.0097039)

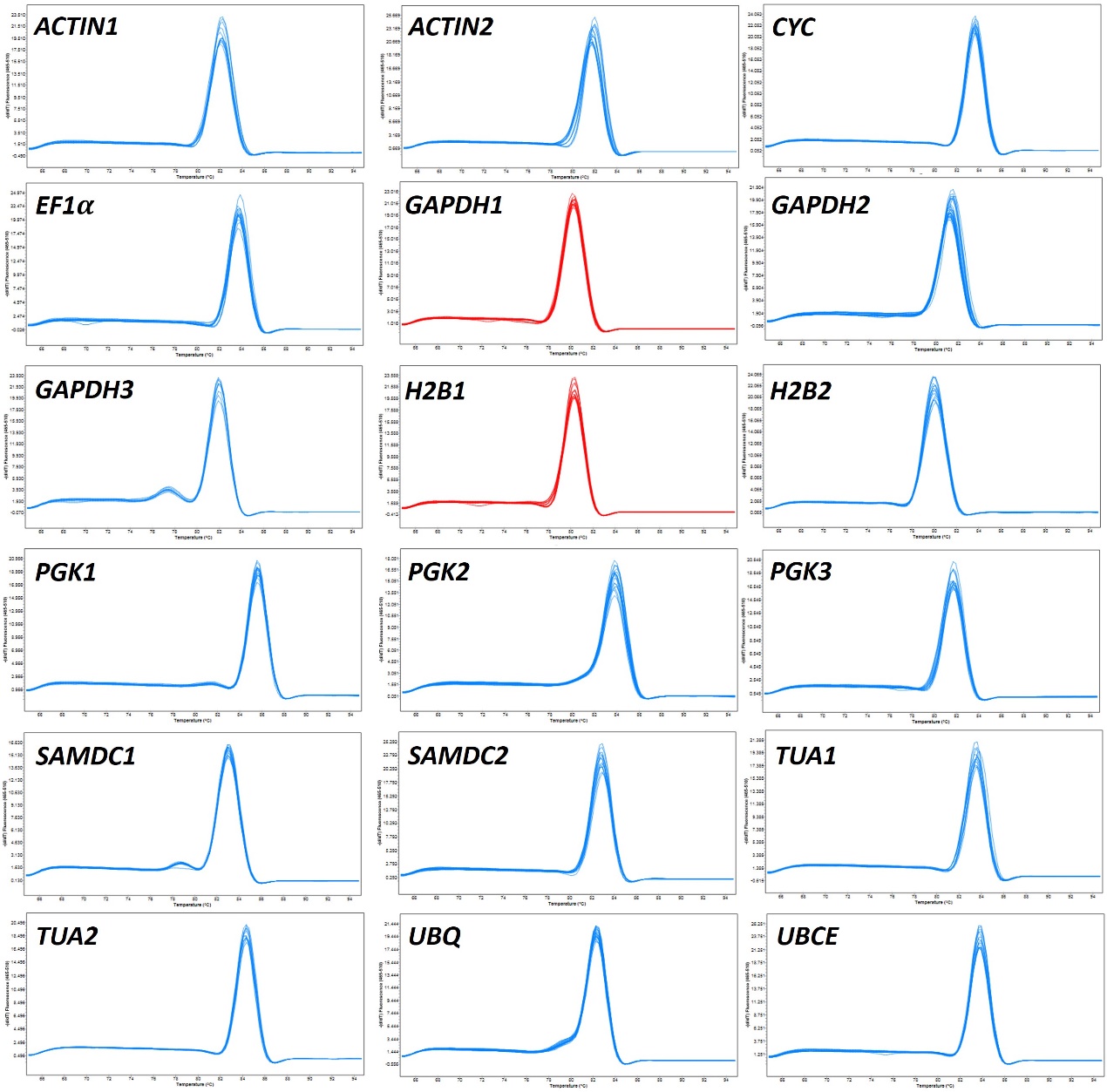


**Figure S3 Dissociation curves for the eighteen reference genes tested in this study.**

Supplement: Figure S3 — Dissociation curves for the eighteen reference genes tested in this study. (DOC) [file pone.0097039.s003.doc]
