# Supplementary material for: Identification and Validation of Reference Genes for Quantitative Real-Time PCR Normalization and Its Applications in Lycium
Source: PLoS One. 2014 May 8;9(5):e97039. doi: 10.1371/journal.pone.0097039 (PMC4014596; doi:10.1371/journal.pone.0097039)
Supplement: Figure S4 — The expression level of F3 ′ 5 ′ H relative to F3 ′ H in L. ruthenicum fruits. The expression ratio of F3′5′Hs/F3′Hs was quantitatively evaluated by qRT-PCR using primers designed on the basis of functionally conserved domains in F3′5′H or F3′H protein while the expression ratio of F3′5′H1/F3′H1 was quantitatively estimated by qRT-PCR using primers designed across 3′ untranslated region and coding region of F3′5′H1 or F3′H1. (DOC) [file pone.0097039.s004.doc]

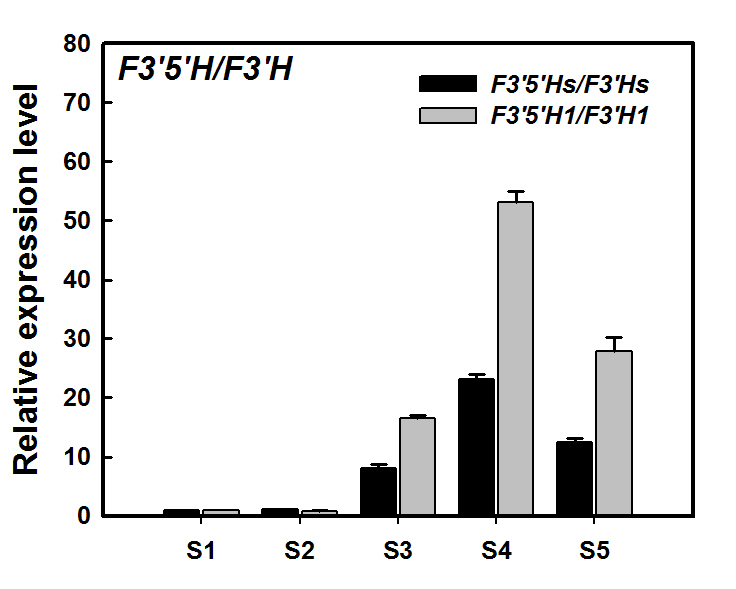


**Figure S4 The expression level of *F3’5’H* relative to *F3’H* in *L. ruthenicum* fruits.** The expression ratio of *F3’5’Hs/F3’Hs* was quantitatively evaluated by qRT-PCR using primers designed on the basis of functionally conserved domains in F3’5’H or F3’H protein while the expression ratio of *F3’5’H1/F3’H1* was quantitatively estimated by qRT-PCR using primers designed across 3’ untranslated region and coding region of *F3’5’H1* or *F3’H1*.
