# Supplementary material for: Identification and Validation of Reference Genes for Quantitative Real-Time PCR Normalization and Its Applications in Lycium
Source: PLoS One. 2014 May 8;9(5):e97039. doi: 10.1371/journal.pone.0097039 (PMC4014596; doi:10.1371/journal.pone.0097039)
Supplement: Table S1 — Experimental design to identify reference genes in Lycium species. Note: TRG, Target of Reference Genes, *indicated that the number of biological replicates. (DOC) [file pone.0097039.s005.doc]

Table S1 **Experimental design to identify reference genes in *Lycium* species**

| Experiments | | |  | Samples | |  | Genes | |  | Species |
| --- | --- | --- | --- | --- | --- | --- | --- | --- | --- | --- |
| ID | Object | |  | Number(*) | Tissue |  | Number | Name |  |
| I | | Identification of TRG stably expressed in different tissues |  | 7(3) | Stem, Leaf, Root, Sepal, Petal, Stamen, Pistil |  | 17 | ACTIN1,ACTIN2,EF1α,GAPDH1,GAPDH2,GAPDH3,UBQ,SAMDC1,  SAMDC2,H2B1,H2B2,PGK1,PGK2,CYC,TUC1,TUC2,UBCE |  | *L. ruthenicum* |
| IIa | | Identification of TRG responding to salt stress |  | 8(3) | Leaf |  | 18 | ACTIN1,ACTIN2,EF1α,GAPDH1,GAPDH2,GAPDH3,UBQ,SAMDC1,  SAMDC2,H2B1,H2B2,PGK1,PGK2,PGK3,CYC,TUA1,TUA2,UBCE |  |
| IIb | | Identification of TRG responding to salt stress |  | 8(3) | Root |  | 18 | ACTIN1,ACTIN2,EF1α,GAPDH1,GAPDH2,GAPDH3,UBQ,SAMDC1,  SAMDC2,H2B1,H2B2,PGK1,PGK2,PGK3,CYC,TUA1,TUA2,UBCE |  |
| IIc | | Identification of TRG responding to salt stress |  | 8(3) | Stem |  | 18 | ACTIN1,ACTIN2,EF1α,GAPDH1,GAPDH2,GAPDH3,UBQ,SAMDC1,  SAMDC2,H2B1,H2B2,PGK1,PGK2,PGK3,CYC,TUA1,TUA2,UBCE |  |
| III | | Identification of TRG stably expressed in developmental fruits |  | 5(3) | Fruit |  | 17 | ACTIN1,ACTIN2,EF1α,GAPDH1,GAPDH2,GAPDH3,UBQ,SAMDC1,  SAMDC2,H2B1,H2B2,PGK1,PGK2,CYC,TUA1,TUA2,UBCE |  |
| IV | | Identification of TRG stably expressed in developmental fruits |  | 5(3) | Fruit |  | 12 | ACTIN1,ACTIN2,EF1α,GAPDH1,GAPDH2,UBQ,SAMDC1,SAMDC2  ,H2B1,H2B2,PGK2,TUA2 |  | *L. barbarum* |
| V | | Identification of TRG responding to salt stress |  | 7(3) | Root |  | 9 | ACTIN1,ACTIN2,GAPDH1,UBQ,SAMDC1,SAMDC2,H2B1,H2B2,  PGK2 |  |

Note: TRG, Target of Reference Genes, * indicated that the number of biological replicates.
