# Supplementary material for: Identification and Validation of Reference Genes for Quantitative Real-Time PCR Normalization and Its Applications in Lycium
Source: PLoS One. 2014 May 8;9(5):e97039. doi: 10.1371/journal.pone.0097039 (PMC4014596; doi:10.1371/journal.pone.0097039)
Supplement: Table S2 — Annotation of Lycium HKGs. (DOC) [file pone.0097039.s006.doc]

**Table S2 Annotation of *Lycium*** HKGs

| Gene | Subject Genes/Accession | Species | E-Value | Annotation |
| --- | --- | --- | --- | --- |
| *ACTIN1* | *actin3*/EOY07537 | *Theobroma cacao* | 0 | actin |
| *ACTIN2* | *actin*/BAD27408 | *Nicotiana tabacum* | 0 | actin |
| *EF1α* | *EF1α*/XP_003629885 | *Medicago truncatula* | 3.00E-49 | Elongation factor 1-alpha |
| *GAPDH1* | *GAPDH*/XP_004242576 | *Solanum lycopersicum* | 0 | glyceraldehyde-3-phosphate dehydrogenase-like |
| *GAPDH2* | *GAPDH*/CBL43264 | *Solanum tuberosum* | 0 | glyceraldehyde-3-phosphate dehydrogenase |
| *GAPDH3* | *GAPDH*/XP_004231523 | *S. lycopersicum* | 0 | glyceraldehyde-3-phosphate dehydrogenase A |
| *UBQ* | *UBQ*/ABK42077 | *Capsicum annuum* | 4.00E-90 | ubiquitin extension protein |
| *SAMDC1* | *SAMDC2*/ABY55855 | *S. lycopersicum* | 0 | S-adenosylmethionine decarboxylase 2 |
| *SAMDC2* | *SAMDC*/AAB88854 | *N. tabacum* | 0 | S-adenosylmethionine decarboxylase |
| *H2B1* | *H2B*/XP_003625510 | *M. truncatula* | 6.00E-58 | histone H2B |
| *H2B2* | *H2B*/ABB72812 | *S. tuberosum* | 2.00E-59 | histone H2B-like protein |
| *PGK1* | *PGK*/Q42962 | *N. tabacum* | 0 | phosphoglycerate kinase |
| *PGK2* | *PGK*/XP_004243968 | *S. lycopersicum* | 0 | phosphoglycerate kinase |
| *PGK3* | *PGK*/Q42962 | *N. tabacum* | 1.00E-158 | phosphoglycerate kinase |
| *CYC* | *CYC*/ACB05668 | *C. annuum* | 8.00E-105 | cyclophilin |
| *TUA1* | *TUA*/CAD13177 | *N. tabacum* | 0 | alpha-tubulin |
| *TUA2* | *TUA*/CAD13178 | *N. tabacum* | 0 | alpha-tubulin |
| *UBCE* | *UBCE*/NP_001234557 | *S. lycopersicum* | 4.00E-145 | ubiquitin conjugating enzyme 2 |
