# Supplementary material for: Identification and Validation of Reference Genes for Quantitative Real-Time PCR Normalization and Its Applications in Lycium
Source: PLoS One. 2014 May 8;9(5):e97039. doi: 10.1371/journal.pone.0097039 (PMC4014596; doi:10.1371/journal.pone.0097039)
Supplement: Table S3 — qRT-PCR primer for genes used to validate target of reference genes. (DOC) [file pone.0097039.s007.doc]

Table S3 qRT-PCR primer for genes used to validate target of reference genes

| Gene | Primer Name | Sequence(5'---3') |
| --- | --- | --- |
| *LrNAC* | NAC-RT-F | GCAACAACAAAATTGCAACA |
|  | NAC-RT-R | CCACCAAATTTGACTTGCATT |
| *LrPG* | PG-RT-F | TCACCACATTGAACTGAAGGA |
|  | PG-RT-R | GAAGACCCTCGACTCCAACA |
| *LrF3'5'Hs* | F3’5’Hs-RT-F | TGCAGGACGAAGAATTTGTG |
|  | F3’5’Hs-RT-F | CCAAAGGTAGCCTTGGAGTAA |
| *LrF3'5'H1* | F3’5’H1-RT-F | TGGGAACTTTGGTTCATTCA |
|  | F3’5’H1-RT-R | TAGCCAAATCGACTCCCAAA |
| *LrF3'Hs* | F3’Hs-RT-F | TGGCAATCTTTGCTCTAATTCT |
|  | F3’Hs-RT-R | ACCCGAAGCTGCAACCAC |
| *LrF3'H1* | F3’H1-RT-F | AACATGGAGGAAGCATTTGG |
|  | F3’H1-RT-R | TGGCCAGATGTGTTAAAAGC |
| *LrAN2* | AN2-RT-F | AATTTCACCACCCATGCAAC |
|  | AN2-RT-R | TTTGGGATACAAAGTACAAACAACTT |
| *LrAN1b* | AN1b-RT-F | GAAAGCGAGCATTCTGGAAG |
|  | AN1b-RT-R | ATCACACACATCGTCGGTTG |
| *LrAN11* | AN11-RT-F | CTGCGACACGTGATTGGAT |
|  | AN11-RT-R | CCAAGCTTTAACCCCTTTCC |
| *LrJAF13* | JAF13-RT-F2 | GGATTGCCATACCGTTCAGT |
|  | JAF13-RT-R2 | ATTGACCAACCTTCGGTGAC |
